# Supplementary figures and images for: Food and light availability induce plastic responses in fire salamander larvae from contrasting environments
Source: PeerJ. 2023 Oct 4;11:e16046. doi: 10.7717/peerj.16046 (PMC10559897; doi:10.7717/peerj.16046)

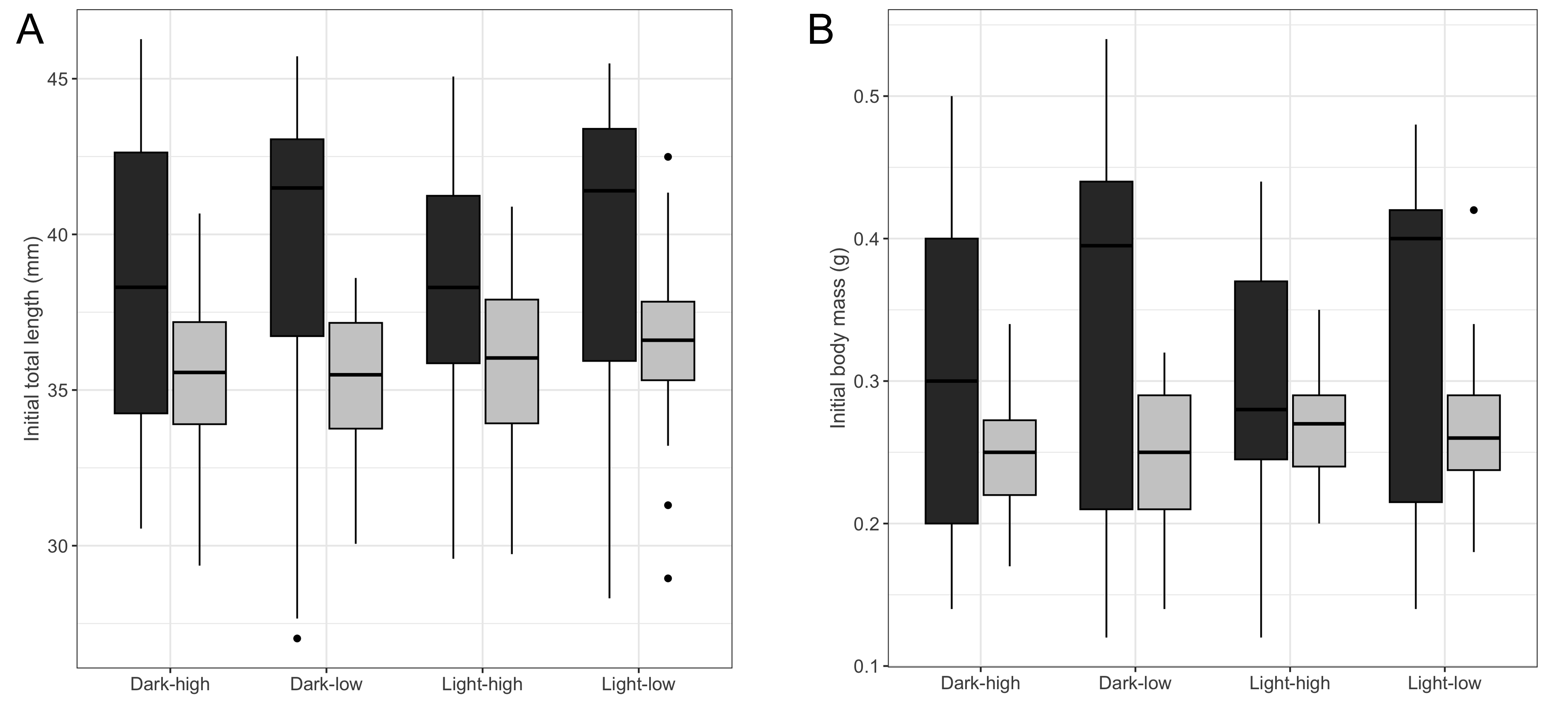

Supplement: Supplemental Information 1 — (A) initial total length and (B) initial body mass, across treatments. Treatments are noted as low versus high for food availability, and light versus dark for the 8-hours and 0-hours photoperiod, respectively. Boxplots represent the median (dark midline) and spread of values, with boxes encompassing the 25th and 75th percentiles, and the whiskers an additional 1.5 of the interquartile range. The closed circles outside the boxplot correspond to outliers. Dark gray boxplots represent larvae from subterranean habitats, while the light gray boxplots represent larvae from the surface. [file peerj-11-16046-s001.jpg]

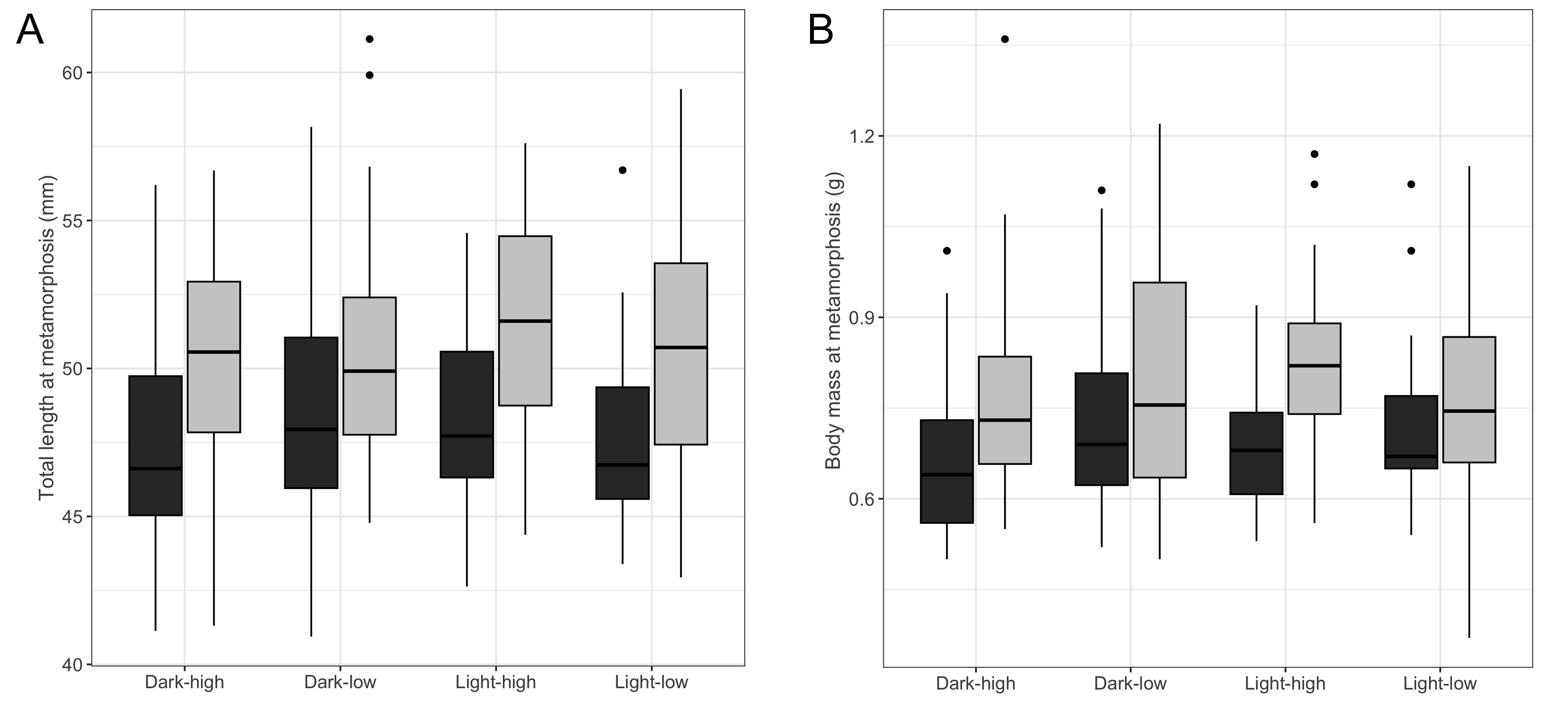

Supplement: Supplemental Information 2 — (A) total length and (B) body mass, across treatments. Treatments are noted as low versus high for food availability, and light versus dark for the 8-hours and 0-hours photoperiod, respectively. Boxplots represent the median (dark midline) and spread of values, with boxes encompassing the 25th and 75th percentiles, and the whiskers an additional 1.5 of the interquartile range. The closed circles outside the boxplot correspond to outliers. Dark gray boxplots represent larvae from subterranean habitats, while the light gray boxplots represent larvae from the surface. [file peerj-11-16046-s002.jpg]
