# Supplementary material for: Food and light availability induce plastic responses in fire salamander larvae from contrasting environments
Source: PeerJ. 2023 Oct 4;11:e16046. doi: 10.7717/peerj.16046 (PMC10559897; doi:10.7717/peerj.16046)
Supplement: Supplemental Information 3 — The four experimental treatments were: high food availability and 8-hour light (high-light), low food availability and 8-hour light (low-light), high food availability and 0-hour light (high-dark), low food availability and 0-hour light (low-dark). n = sample size. [file peerj-11-16046-s003.docx]

Table S1. Assignment of 260 *Salamandra salamandra* larvae (developmental stage 1) from each habitat type and population, by year and experimental treatment. The four experimental treatments were: high food availability and 8-hour light (high-light), low food availability and 8-hour light (low-light), high food availability and 0-hour light (high-dark), low food availability and 0-hour light (low-dark). *n* = sample size.

| Treatment | Population | Habitat type | *n* | year |
| --- | --- | --- | --- | --- |
| high-light | Iconie | surface | 32 | 2016 |
| low-light | Iconie | surface | 32 | 2016 |
| high-dark | Iconie | surface | 32 | 2016 |
| low-dark | Iconie | surface | 32 | 2016 |
| high-light | Iconie | surface | 14 | 2017 |
| low-light | Iconie | surface | 14 | 2017 |
| high-dark | Iconie | surface | 14 | 2017 |
| low-dark | Iconie | surface | 14 | 2017 |
| high-light | Buzau | subterranean | 5 | 2017 |
| low-light | Buzau | subterranean | 7 | 2017 |
| high-dark | Buzau | subterranean | 8 | 2017 |
| low-dark | Buzau | subterranean | 9 | 2017 |
| high-light | Gaura cu Musca | subterranean | 14 | 2017 |
| low-light | Gaura cu Musca | subterranean | 11 | 2017 |
| high-dark | Gaura cu Musca | subterranean | 12 | 2017 |
| low-dark | Gaura cu Musca | subterranean | 10 | 2017 |
