# Supplementary material for: Food and light availability induce plastic responses in fire salamander larvae from contrasting environments
Source: PeerJ. 2023 Oct 4;11:e16046. doi: 10.7717/peerj.16046 (PMC10559897; doi:10.7717/peerj.16046)
Supplement: Supplemental Information 4 — Photoperiod = light (8-hours photoperiod), dark (0-hours photoperiod), Food = high (high food availability), low (low food availability). Time_met = time to metamorphosis (days), BM_0 = initial body mass (g), BM_met = body mass at metamorphosis (g), TL_0 = initial total length (mm), TL_met = total length at metamorphosis (mm), Growth_BM = body mass growth rate (g/day), and Growth_TL = total length growth rate (mm/day). [file peerj-11-16046-s004.docx]

Table S2. Dataset used for the analyses of 195 *Salamandra salamandra* larvae which completed metamorphosis by the end of the experiment. Photoperiod = light (8-hours photoperiod), dark (0-hours photoperiod), Food = high (high food availability), low (low food availability). Time_met_ = time to metamorphosis (days), BM_0_ = initial body mass (g), BM_met_ = body mass at metamorphosis (g), TL_0_ = initial total length (mm), TL_met_ = total length at metamorphosis (mm), Growth_BM_ = body mass growth rate (g/day), and Growth_TL_ = total length growth rate (mm/day).

| Photoperiod | Food | Time_met_ | BM_0_ | BM_met_ | TL_0_ | TL_met_ | Growth_BM_ | Growth_TL_ | Habitat | Year | Population |
| --- | --- | --- | --- | --- | --- | --- | --- | --- | --- | --- | --- |
| dark | high | 153 | 0.2 | 1.01 | 34.52 | 56.2 | 0.005 | 0.142 | subterranean | 2017 | Buzau |
| dark | high | 76 | 0.31 | 0.56 | 36.95 | 45.04 | 0.003 | 0.107 | subterranean | 2017 | Buzau |
| dark | high | 106 | 0.22 | 0.94 | 34.97 | 52.41 | 0.007 | 0.164 | subterranean | 2017 | Buzau |
| dark | high | 121 | 0.2 | 0.82 | 34.25 | 54.5 | 0.005 | 0.167 | subterranean | 2017 | Buzau |
| dark | high | 91 | 0.25 | 0.78 | 39.68 | 54.34 | 0.006 | 0.161 | subterranean | 2017 | Buzau |
| dark | high | 121 | 0.21 | 0.73 | 34.09 | 49.57 | 0.004 | 0.128 | subterranean | 2017 | Buzau |
| dark | low | 136 | 0.25 | 1.11 | 40.04 | 58.16 | 0.006 | 0.133 | subterranean | 2017 | Buzau |
| dark | low | 106 | 0.35 | 0.72 | 40.58 | 47.9 | 0.003 | 0.069 | subterranean | 2017 | Buzau |
| dark | low | 167 | 0.21 | 0.92 | 35.3 | 50.95 | 0.004 | 0.094 | subterranean | 2017 | Buzau |
| dark | low | 167 | 0.17 | 0.96 | 33.59 | 57.31 | 0.005 | 0.142 | subterranean | 2017 | Buzau |
| dark | low | 136 | 0.21 | 0.77 | 37.21 | 51.33 | 0.004 | 0.104 | subterranean | 2017 | Buzau |
| dark | low | 136 | 0.44 | 1.08 | 43.28 | 56.95 | 0.005 | 0.1 | subterranean | 2017 | Buzau |
| dark | high | 136 | 0.14 | 0.55 | 30.55 | 42.25 | 0.003 | 0.086 | subterranean | 2017 | Gaura cu Musca |
| dark | high | 91 | 0.14 | 0.56 | 31.17 | 42.08 | 0.005 | 0.12 | subterranean | 2017 | Gaura cu Musca |
| dark | high | 106 | 0.15 | 0.5 | 32.75 | 41.13 | 0.003 | 0.079 | subterranean | 2017 | Gaura cu Musca |
| dark | high | 76 | 0.4 | 0.62 | 42.63 | 46.37 | 0.003 | 0.049 | subterranean | 2017 | Gaura cu Musca |
| dark | high | 76 | 0.38 | 0.63 | 41.72 | 45.83 | 0.003 | 0.054 | subterranean | 2017 | Gaura cu Musca |
| dark | high | 76 | 0.5 | 0.64 | 46.27 | 46.75 | 0.002 | 0.006 | subterranean | 2017 | Gaura cu Musca |
| dark | high | 91 | 0.3 | 0.71 | 38.3 | 45.38 | 0.005 | 0.078 | subterranean | 2017 | Gaura cu Musca |
| dark | high | 76 | 0.41 | 0.59 | 42.29 | 46.62 | 0.002 | 0.057 | subterranean | 2017 | Gaura cu Musca |
| dark | high | 76 | 0.34 | 0.67 | 45.21 | 49.74 | 0.004 | 0.06 | subterranean | 2017 | Gaura cu Musca |
| dark | high | 76 | 0.42 | 0.55 | 42.7 | 44.48 | 0.002 | 0.023 | subterranean | 2017 | Gaura cu Musca |
| dark | high | 76 | 0.41 | 0.7 | 42.8 | 46.74 | 0.004 | 0.052 | subterranean | 2017 | Gaura cu Musca |
| dark | low | 106 | 0.12 | 0.57 | 27.66 | 45.64 | 0.004 | 0.17 | subterranean | 2017 | Gaura cu Musca |
| dark | low | 121 | 0.12 | 0.52 | 27.02 | 40.94 | 0.003 | 0.115 | subterranean | 2017 | Gaura cu Musca |
| dark | low | 91 | 0.42 | 0.71 | 42.98 | 47.79 | 0.003 | 0.053 | subterranean | 2017 | Gaura cu Musca |
| dark | low | 91 | 0.37 | 0.6 | 40.7 | 43.9 | 0.003 | 0.035 | subterranean | 2017 | Gaura cu Musca |
| dark | low | 76 | 0.44 | 0.7 | 42.96 | 48.74 | 0.003 | 0.076 | subterranean | 2017 | Gaura cu Musca |
| dark | low | 76 | 0.44 | 0.59 | 42.69 | 46.06 | 0.002 | 0.044 | subterranean | 2017 | Gaura cu Musca |
| dark | low | 76 | 0.42 | 0.64 | 42.28 | 44.13 | 0.003 | 0.024 | subterranean | 2017 | Gaura cu Musca |
| dark | low | 76 | 0.47 | 0.68 | 44.1 | 49.11 | 0.003 | 0.066 | subterranean | 2017 | Gaura cu Musca |
| dark | low | 91 | 0.52 | 0.67 | 44.26 | 47.63 | 0.002 | 0.037 | subterranean | 2017 | Gaura cu Musca |
| dark | low | 76 | 0.54 | 0.63 | 45.72 | 47.98 | 0.001 | 0.03 | subterranean | 2017 | Gaura cu Musca |
| dark | high | 106 | 0.19 | 0.64 | 35.09 | 50.43 | 0.004 | 0.145 | surface | 2016 | Iconie |
| dark | high | 106 | 0.25 | 0.95 | 34.52 | 51.14 | 0.007 | 0.157 | surface | 2016 | Iconie |
| dark | high | 106 | 0.25 | 1.06 | 35.44 | 54.33 | 0.008 | 0.178 | surface | 2016 | Iconie |
| dark | high | 101 | 0.22 | 0.55 | 34.72 | 45.72 | 0.003 | 0.109 | surface | 2016 | Iconie |
| dark | high | 93 | 0.17 | 0.71 | 32.83 | 52.51 | 0.006 | 0.212 | surface | 2016 | Iconie |
| dark | high | 100 | 0.23 | 0.68 | 29.46 | 48.27 | 0.005 | 0.188 | surface | 2016 | Iconie |
| dark | high | 160 | 0.22 | 1.36 | 29.36 | 56.69 | 0.007 | 0.171 | surface | 2016 | Iconie |
| dark | high | 100 | 0.26 | 0.85 | 32.02 | 50.98 | 0.006 | 0.19 | surface | 2016 | Iconie |
| dark | high | 97 | 0.25 | 0.75 | 35.51 | 53.15 | 0.005 | 0.182 | surface | 2016 | Iconie |
| dark | high | 80 | 0.25 | 0.72 | 36.68 | 50.68 | 0.006 | 0.175 | surface | 2016 | Iconie |
| dark | high | 93 | 0.26 | 0.78 | 36.68 | 52.37 | 0.006 | 0.169 | surface | 2016 | Iconie |
| dark | high | 100 | 0.19 | 0.81 | 34.16 | 52.92 | 0.006 | 0.188 | surface | 2016 | Iconie |
| dark | high | 93 | 0.23 | 0.91 | 32.42 | 50.71 | 0.007 | 0.197 | surface | 2016 | Iconie |
| dark | high | 121 | 0.2 | 1.07 | 33.85 | 55.07 | 0.007 | 0.175 | surface | 2016 | Iconie |
| dark | high | 97 | 0.2 | 0.64 | 33 | 47.45 | 0.005 | 0.149 | surface | 2016 | Iconie |
| dark | high | 93 | 0.27 | 0.65 | 35.67 | 52.97 | 0.004 | 0.186 | surface | 2016 | Iconie |
| dark | high | 106 | 0.2 | 0.82 | 31.81 | 48.05 | 0.006 | 0.153 | surface | 2016 | Iconie |
| dark | high | 93 | 0.28 | 0.69 | 37.56 | 54.43 | 0.004 | 0.181 | surface | 2016 | Iconie |
| dark | high | 89 | 0.25 | 1.06 | 40.67 | 54.23 | 0.009 | 0.152 | surface | 2016 | Iconie |
| dark | high | 93 | 0.25 | 0.73 | 39.67 | 51.62 | 0.005 | 0.129 | surface | 2016 | Iconie |
| dark | high | 100 | 0.21 | 0.71 | 34.14 | 48.71 | 0.005 | 0.146 | surface | 2016 | Iconie |
| dark | low | 94 | 0.29 | 0.61 | 36.55 | 48.27 | 0.003 | 0.125 | surface | 2016 | Iconie |
| dark | low | 140 | 0.2 | 0.84 | 34.46 | 51.26 | 0.005 | 0.12 | surface | 2016 | Iconie |
| dark | low | 147 | 0.3 | 0.95 | 38.3 | 52.25 | 0.004 | 0.095 | surface | 2016 | Iconie |
| dark | low | 80 | 0.25 | 0.52 | 34.43 | 45.37 | 0.003 | 0.137 | surface | 2016 | Iconie |
| dark | low | 106 | 0.28 | 0.61 | 36.02 | 49.57 | 0.003 | 0.128 | surface | 2016 | Iconie |
| dark | low | 119 | 0.26 | 0.75 | 34.12 | 48.81 | 0.004 | 0.123 | surface | 2016 | Iconie |
| dark | low | 142 | 0.21 | 0.98 | 34.85 | 55.74 | 0.005 | 0.147 | surface | 2016 | Iconie |
| dark | low | 100 | 0.24 | 0.65 | 33.34 | 46.03 | 0.004 | 0.127 | surface | 2016 | Iconie |
| dark | low | 137 | 0.27 | 1.05 | 35.25 | 61.13 | 0.006 | 0.189 | surface | 2016 | Iconie |
| dark | low | 151 | 0.2 | 0.7 | 33.64 | 52.45 | 0.003 | 0.125 | surface | 2016 | Iconie |
| dark | low | 164 | 0.24 | 1.2 | 37.82 | 59.91 | 0.006 | 0.135 | surface | 2016 | Iconie |
| dark | low | 168 | 0.16 | 1.09 | 30.47 | 52.71 | 0.006 | 0.132 | surface | 2016 | Iconie |
| dark | low | 132 | 0.18 | 0.84 | 32.25 | 50.59 | 0.005 | 0.139 | surface | 2016 | Iconie |
| dark | low | 168 | 0.21 | 1.05 | 30.06 | 50.91 | 0.005 | 0.124 | surface | 2016 | Iconie |
| dark | low | 168 | 0.27 | 1.11 | 34.6 | 56.82 | 0.005 | 0.132 | surface | 2016 | Iconie |
| dark | low | 160 | 0.24 | 1.22 | 32.43 | 55.54 | 0.006 | 0.144 | surface | 2016 | Iconie |
| dark | low | 147 | 0.32 | 0.86 | 36.58 | 51.86 | 0.004 | 0.104 | surface | 2016 | Iconie |
| dark | low | 140 | 0.16 | 0.76 | 32.59 | 49.26 | 0.004 | 0.119 | surface | 2016 | Iconie |
| dark | low | 94 | 0.3 | 0.63 | 36.94 | 50.25 | 0.004 | 0.142 | surface | 2016 | Iconie |
| dark | low | 93 | 0.3 | 0.69 | 37.92 | 50.37 | 0.004 | 0.134 | surface | 2016 | Iconie |
| dark | low | 119 | 0.25 | 0.72 | 35.73 | 48.73 | 0.004 | 0.109 | surface | 2016 | Iconie |
| dark | high | 91 | 0.32 | 0.81 | 37.09 | 48.08 | 0.005 | 0.121 | surface | 2017 | Iconie |
| dark | high | 106 | 0.34 | 0.86 | 38.23 | 53.78 | 0.005 | 0.147 | surface | 2017 | Iconie |
| dark | high | 91 | 0.25 | 0.83 | 37.46 | 48.26 | 0.006 | 0.119 | surface | 2017 | Iconie |
| dark | high | 91 | 0.3 | 0.78 | 37.87 | 46.89 | 0.005 | 0.099 | surface | 2017 | Iconie |
| dark | high | 91 | 0.26 | 0.71 | 35.62 | 47.97 | 0.005 | 0.136 | surface | 2017 | Iconie |
| dark | high | 76 | 0.3 | 0.62 | 36.64 | 45.06 | 0.004 | 0.111 | surface | 2017 | Iconie |
| dark | high | 106 | 0.3 | 0.73 | 36.36 | 49.05 | 0.004 | 0.12 | surface | 2017 | Iconie |
| dark | high | 91 | 0.26 | 0.6 | 37.96 | 44.22 | 0.004 | 0.069 | surface | 2017 | Iconie |
| dark | high | 91 | 0.29 | 0.66 | 36.39 | 41.31 | 0.004 | 0.054 | surface | 2017 | Iconie |
| dark | high | 91 | 0.26 | 0.65 | 33.92 | 45.16 | 0.004 | 0.124 | surface | 2017 | Iconie |
| dark | high | 106 | 0.3 | 0.65 | 37.82 | 45.08 | 0.003 | 0.068 | surface | 2017 | Iconie |
| dark | low | 167 | 0.21 | 0.96 | 37.37 | 54.64 | 0.004 | 0.103 | surface | 2017 | Iconie |
| dark | low | 136 | 0.14 | 0.87 | 31.49 | 48.38 | 0.005 | 0.124 | surface | 2017 | Iconie |
| dark | low | 106 | 0.29 | 0.71 | 38.41 | 46.81 | 0.004 | 0.079 | surface | 2017 | Iconie |
| dark | low | 106 | 0.29 | 0.8 | 37.23 | 48.58 | 0.005 | 0.107 | surface | 2017 | Iconie |
| dark | low | 91 | 0.27 | 0.5 | 35.8 | 44.83 | 0.003 | 0.099 | surface | 2017 | Iconie |
| dark | low | 136 | 0.21 | 0.61 | 34.84 | 46.13 | 0.003 | 0.083 | surface | 2017 | Iconie |
| dark | low | 106 | 0.29 | 0.71 | 38.44 | 47.59 | 0.004 | 0.086 | surface | 2017 | Iconie |
| dark | low | 106 | 0.24 | 0.63 | 35.97 | 44.78 | 0.004 | 0.083 | surface | 2017 | Iconie |
| dark | low | 91 | 0.29 | 0.63 | 38.6 | 45.15 | 0.004 | 0.072 | surface | 2017 | Iconie |
| light | high | 121 | 0.24 | 0.83 | 38.98 | 54.58 | 0.005 | 0.129 | subterranean | 2017 | Buzau |
| light | high | 153 | 0.13 | 0.85 | 29.58 | 52.85 | 0.005 | 0.152 | subterranean | 2017 | Buzau |
| light | high | 121 | 0.26 | 0.92 | 35.73 | 52.73 | 0.005 | 0.141 | subterranean | 2017 | Buzau |
| light | low | 91 | 0.34 | 0.74 | 40.06 | 49.03 | 0.004 | 0.099 | subterranean | 2017 | Buzau |
| light | low | 121 | 0.22 | 0.87 | 37.1 | 49.7 | 0.005 | 0.104 | subterranean | 2017 | Buzau |
| light | low | 153 | 0.21 | 1.12 | 35.58 | 56.7 | 0.006 | 0.138 | subterranean | 2017 | Buzau |
| light | low | 153 | 0.18 | 1.01 | 28.31 | 52.57 | 0.005 | 0.159 | subterranean | 2017 | Buzau |
| light | low | 121 | 0.31 | 0.67 | 36.29 | 51.64 | 0.003 | 0.127 | subterranean | 2017 | Buzau |
| light | high | 76 | 0.15 | 0.53 | 31.89 | 42.63 | 0.005 | 0.141 | subterranean | 2017 | Gaura cu Musca |
| light | high | 106 | 0.12 | 0.75 | 29.86 | 48.29 | 0.006 | 0.174 | subterranean | 2017 | Gaura cu Musca |
| light | high | 76 | 0.29 | 0.72 | 37.61 | 47.83 | 0.006 | 0.135 | subterranean | 2017 | Gaura cu Musca |
| light | high | 76 | 0.36 | 0.63 | 41.35 | 45.8 | 0.004 | 0.059 | subterranean | 2017 | Gaura cu Musca |
| light | high | 91 | 0.27 | 0.6 | 37.55 | 48.82 | 0.004 | 0.124 | subterranean | 2017 | Gaura cu Musca |
| light | high | 91 | 0.26 | 0.58 | 36.25 | 45.97 | 0.004 | 0.107 | subterranean | 2017 | Gaura cu Musca |
| light | high | 76 | 0.44 | 0.72 | 45.07 | 51.15 | 0.004 | 0.08 | subterranean | 2017 | Gaura cu Musca |
| light | high | 91 | 0.37 | 0.64 | 40.91 | 47.36 | 0.003 | 0.071 | subterranean | 2017 | Gaura cu Musca |
| light | high | 91 | 0.37 | 0.57 | 40.34 | 47.35 | 0.002 | 0.077 | subterranean | 2017 | Gaura cu Musca |
| light | high | 76 | 0.42 | 0.69 | 43.41 | 45.59 | 0.004 | 0.029 | subterranean | 2017 | Gaura cu Musca |
| light | high | 76 | 0.44 | 0.67 | 44.31 | 47.6 | 0.003 | 0.043 | subterranean | 2017 | Gaura cu Musca |
| light | low | 91 | 0.15 | 0.56 | 31.61 | 43.39 | 0.005 | 0.129 | subterranean | 2017 | Gaura cu Musca |
| light | low | 106 | 0.14 | 0.71 | 30.51 | 46.74 | 0.005 | 0.153 | subterranean | 2017 | Gaura cu Musca |
| light | low | 91 | 0.4 | 0.66 | 42.38 | 45.31 | 0.003 | 0.032 | subterranean | 2017 | Gaura cu Musca |
| light | low | 91 | 0.41 | 0.78 | 41.4 | 43.74 | 0.004 | 0.026 | subterranean | 2017 | Gaura cu Musca |
| light | low | 76 | 0.41 | 0.65 | 42.95 | 46.05 | 0.003 | 0.041 | subterranean | 2017 | Gaura cu Musca |
| light | low | 76 | 0.47 | 0.54 | 43.08 | 46.18 | 0.001 | 0.041 | subterranean | 2017 | Gaura cu Musca |
| light | low | 91 | 0.43 | 0.76 | 44.94 | 48.41 | 0.004 | 0.038 | subterranean | 2017 | Gaura cu Musca |
| light | low | 91 | 0.42 | 0.67 | 43.7 | 45.02 | 0.003 | 0.014 | subterranean | 2017 | Gaura cu Musca |
| light | low | 76 | 0.42 | 0.65 | 44.1 | 45.87 | 0.003 | 0.023 | subterranean | 2017 | Gaura cu Musca |
| light | low | 76 | 0.48 | 0.61 | 45.49 | 47.94 | 0.002 | 0.032 | subterranean | 2017 | Gaura cu Musca |
| light | high | 86 | 0.28 | 0.66 | 37.77 | 46.73 | 0.004 | 0.104 | surface | 2016 | Iconie |
| light | high | 89 | 0.27 | 0.81 | 29.85 | 53.63 | 0.006 | 0.267 | surface | 2016 | Iconie |
| light | high | 102 | 0.27 | 0.92 | 35.15 | 54.57 | 0.006 | 0.19 | surface | 2016 | Iconie |
| light | high | 102 | 0.23 | 0.76 | 34.97 | 54.37 | 0.005 | 0.19 | surface | 2016 | Iconie |
| light | high | 106 | 0.25 | 0.69 | 32.17 | 48.5 | 0.004 | 0.154 | surface | 2016 | Iconie |
| light | high | 97 | 0.3 | 0.87 | 35.85 | 57.01 | 0.006 | 0.218 | surface | 2016 | Iconie |
| light | high | 100 | 0.27 | 0.82 | 36.05 | 56.19 | 0.006 | 0.201 | surface | 2016 | Iconie |
| light | high | 106 | 0.24 | 0.85 | 33.3 | 50.12 | 0.006 | 0.159 | surface | 2016 | Iconie |
| light | high | 100 | 0.26 | 0.83 | 35.5 | 53.33 | 0.006 | 0.178 | surface | 2016 | Iconie |
| light | high | 80 | 0.27 | 0.66 | 36.37 | 48.96 | 0.005 | 0.157 | surface | 2016 | Iconie |
| light | high | 91 | 0.29 | 0.95 | 37.93 | 53.45 | 0.007 | 0.171 | surface | 2016 | Iconie |
| light | high | 91 | 0.3 | 1 | 39.54 | 57.61 | 0.008 | 0.199 | surface | 2016 | Iconie |
| light | high | 83 | 0.27 | 0.78 | 37.64 | 49.55 | 0.006 | 0.144 | surface | 2016 | Iconie |
| light | high | 91 | 0.28 | 0.83 | 31.47 | 50.54 | 0.006 | 0.21 | surface | 2016 | Iconie |
| light | high | 165 | 0.2 | 1.17 | 39.63 | 51.6 | 0.006 | 0.073 | surface | 2016 | Iconie |
| light | high | 72 | 0.25 | 0.62 | 38.09 | 53.34 | 0.005 | 0.212 | surface | 2016 | Iconie |
| light | high | 83 | 0.33 | 0.87 | 29.73 | 51.03 | 0.007 | 0.257 | surface | 2016 | Iconie |
| light | high | 112 | 0.29 | 1.02 | 37.72 | 57.56 | 0.007 | 0.177 | surface | 2016 | Iconie |
| light | high | 106 | 0.24 | 1.12 | 32.07 | 55.37 | 0.008 | 0.22 | surface | 2016 | Iconie |
| light | high | 137 | 0.28 | 0.91 | 32.31 | 56.35 | 0.005 | 0.175 | surface | 2016 | Iconie |
| light | high | 102 | 0.22 | 0.67 | 34.72 | 52.14 | 0.004 | 0.171 | surface | 2016 | Iconie |
| light | high | 103 | 0.21 | 0.79 | 32.45 | 52.6 | 0.006 | 0.196 | surface | 2016 | Iconie |
| light | high | 83 | 0.27 | 0.84 | 37.88 | 52.14 | 0.007 | 0.172 | surface | 2016 | Iconie |
| light | high | 102 | 0.27 | 1.01 | 36.03 | 55.71 | 0.007 | 0.193 | surface | 2016 | Iconie |
| light | high | 91 | 0.3 | 0.86 | 39.64 | 49.35 | 0.006 | 0.107 | surface | 2016 | Iconie |
| light | high | 86 | 0.32 | 0.8 | 39.82 | 55.38 | 0.006 | 0.181 | surface | 2016 | Iconie |
| light | low | 154 | 0.24 | 0.75 | 35.62 | 51.83 | 0.003 | 0.105 | surface | 2016 | Iconie |
| light | low | 67 | 0.42 | 0.59 | 33.93 | 48.99 | 0.003 | 0.225 | surface | 2016 | Iconie |
| light | low | 169 | 0.25 | 1.09 | 38.43 | 56.28 | 0.005 | 0.106 | surface | 2016 | Iconie |
| light | low | 142 | 0.22 | 0.92 | 34.79 | 56.48 | 0.005 | 0.153 | surface | 2016 | Iconie |
| light | low | 106 | 0.3 | 0.64 | 38.14 | 46.91 | 0.003 | 0.083 | surface | 2016 | Iconie |
| light | low | 82 | 0.32 | 0.37 | 37.68 | 43.38 | 0.001 | 0.07 | surface | 2016 | Iconie |
| light | low | 128 | 0.2 | 0.72 | 36.46 | 48.08 | 0.004 | 0.091 | surface | 2016 | Iconie |
| light | low | 97 | 0.26 | 0.67 | 36.01 | 51.59 | 0.004 | 0.161 | surface | 2016 | Iconie |
| light | low | 164 | 0.3 | 1.12 | 35.12 | 55.43 | 0.005 | 0.124 | surface | 2016 | Iconie |
| light | low | 140 | 0.18 | 0.86 | 31.3 | 53.42 | 0.005 | 0.158 | surface | 2016 | Iconie |
| light | low | 137 | 0.26 | 0.84 | 37.23 | 54.21 | 0.004 | 0.124 | surface | 2016 | Iconie |
| light | low | 142 | 0.21 | 0.96 | 34.84 | 56.39 | 0.005 | 0.152 | surface | 2016 | Iconie |
| light | low | 154 | 0.23 | 0.93 | 33.21 | 53.15 | 0.005 | 0.129 | surface | 2016 | Iconie |
| light | low | 160 | 0.22 | 0.66 | 35.42 | 45.4 | 0.003 | 0.062 | surface | 2016 | Iconie |
| light | low | 137 | 0.27 | 0.89 | 36.74 | 53.07 | 0.005 | 0.119 | surface | 2016 | Iconie |
| light | low | 93 | 0.32 | 0.55 | 40.46 | 46.9 | 0.002 | 0.069 | surface | 2016 | Iconie |
| light | low | 137 | 0.25 | 0.8 | 38.29 | 52.08 | 0.004 | 0.101 | surface | 2016 | Iconie |
| light | low | 116 | 0.29 | 0.68 | 42.49 | 52.01 | 0.003 | 0.082 | surface | 2016 | Iconie |
| light | low | 119 | 0.2 | 0.7 | 28.95 | 47.71 | 0.004 | 0.158 | surface | 2016 | Iconie |
| light | low | 168 | 0.23 | 1.11 | 36.18 | 57.27 | 0.005 | 0.126 | surface | 2016 | Iconie |
| light | low | 168 | 0.26 | 1.15 | 37.22 | 59.44 | 0.005 | 0.132 | surface | 2016 | Iconie |
| light | low | 160 | 0.25 | 0.81 | 36.46 | 53.09 | 0.004 | 0.104 | surface | 2016 | Iconie |
| light | low | 147 | 0.23 | 0.79 | 34.79 | 54.39 | 0.004 | 0.133 | surface | 2016 | Iconie |
| light | high | 76 | 0.35 | 0.67 | 40.89 | 45.95 | 0.004 | 0.067 | surface | 2017 | Iconie |
| light | high | 106 | 0.24 | 0.91 | 33.85 | 49.18 | 0.006 | 0.145 | surface | 2017 | Iconie |
| light | high | 91 | 0.26 | 0.73 | 37.38 | 44.93 | 0.005 | 0.083 | surface | 2017 | Iconie |
| light | high | 91 | 0.23 | 0.79 | 35.93 | 44.38 | 0.006 | 0.093 | surface | 2017 | Iconie |
| light | high | 76 | 0.32 | 0.82 | 39.69 | 47.66 | 0.007 | 0.105 | surface | 2017 | Iconie |
| light | high | 121 | 0.21 | 0.75 | 34.62 | 48.53 | 0.004 | 0.115 | surface | 2017 | Iconie |
| light | high | 76 | 0.27 | 0.56 | 37.66 | 46.34 | 0.004 | 0.114 | surface | 2017 | Iconie |
| light | high | 91 | 0.22 | 0.64 | 34.01 | 47.18 | 0.005 | 0.145 | surface | 2017 | Iconie |
| light | high | 76 | 0.32 | 0.77 | 37.98 | 49.05 | 0.006 | 0.146 | surface | 2017 | Iconie |
| light | low | 106 | 0.3 | 0.74 | 39.7 | 47.03 | 0.004 | 0.069 | surface | 2017 | Iconie |
| light | low | 106 | 0.28 | 0.66 | 37.78 | 46.25 | 0.004 | 0.08 | surface | 2017 | Iconie |
| light | low | 91 | 0.24 | 0.66 | 36.34 | 47.56 | 0.005 | 0.123 | surface | 2017 | Iconie |
| light | low | 121 | 0.27 | 0.95 | 37.31 | 53.96 | 0.006 | 0.138 | surface | 2017 | Iconie |
| light | low | 106 | 0.25 | 0.74 | 35.52 | 47.03 | 0.005 | 0.109 | surface | 2017 | Iconie |
| light | low | 106 | 0.29 | 0.73 | 38.01 | 43.97 | 0.004 | 0.056 | surface | 2017 | Iconie |
| light | low | 76 | 0.33 | 0.62 | 41.34 | 42.94 | 0.004 | 0.021 | surface | 2017 | Iconie |
| light | low | 106 | 0.29 | 0.81 | 33.99 | 49.64 | 0.005 | 0.148 | surface | 2017 | Iconie |
| light | low | 106 | 0.26 | 0.64 | 39.86 | 51.62 | 0.004 | 0.111 | surface | 2017 | Iconie |
| light | low | 91 | 0.29 | 0.71 | 37.73 | 48.85 | 0.005 | 0.122 | surface | 2017 | Iconie |
| light | low | 91 | 0.34 | 0.8 | 36.89 | 49.83 | 0.005 | 0.142 | surface | 2017 | Iconie |
| light | low | 106 | 0.24 | 0.78 | 35.38 | 48.47 | 0.005 | 0.123 | surface | 2017 | Iconie |
| light | low | 121 | 0.25 | 0.63 | 37.61 | 48.12 | 0.003 | 0.087 | surface | 2017 | Iconie |
