# Supplementary material for: Food and light availability induce plastic responses in fire salamander larvae from contrasting environments
Source: PeerJ. 2023 Oct 4;11:e16046. doi: 10.7717/peerj.16046 (PMC10559897; doi:10.7717/peerj.16046)
Supplement: Supplemental Information 5 [file peerj-11-16046-s005.docx]

Table S3. Summary statistics of initial body mass (g) (BM_0_) and initial total length (mm) (TL_0_) of *Salamandra salamandra* larvae from the three studied populations from Romania. n = sample size; SD = standard deviation.

| Population | Habitat type and sampling year | Size parameter | Mean±SD | min-max |
| --- | --- | --- | --- | --- |
| Iconie (n=184) | surface,  2016-2017 | BM_0_ | 0.26±0.04 | 0.14-0.42 |
|  |  | TL_0_ | 35.79±2.70 | 28.95-42.49 |
| Gaura cu Muscă (n=47) | subterranean, 2017 | BM_0_ | 0.35±0.13 | 0.12-0.54 |
|  |  | TL_0_ | 39.89±5.55 | 27.02-46.27 |
| Buzău (n=29) | subterranean, 2017 | BM_0_ | 0.25±0.07 | 0.13-0.44 |
|  |  | TL_0_ | 36.30±3.61 | 28.31-43.28 |
